# Quantifying the Degree of Movement Dissimilarity between Two Distinct Action Scenarios: An Exploratory Approach with Procrustes Analysis

**DOI:** 10.3389/fpsyg.2017.00640

**Published:** 2017-04-28

**Authors:** Pedro Passos, Tania Campos, Ana Diniz

**Affiliations:** ^1^CIPER, Faculdade de Motricidade Humana/Universidade de LisboaLisboa, Portugal; ^2^Departamento de Fisioterapia, Universidade Federal do Rio Grande do NorteNatal, Brazil

**Keywords:** representativeness, action fidelity, human movement, Procrustes analysis, simulated scenarios

## Abstract

Game consoles allow subjects to perform movements which are visually similar to the movements performed in ‘real’ world scenarios. Beyond entertainment, virtual reality devices are being used in several domains: sports performance; motor rehabilitation; training of risk professions. This article presents the Procrustes method to measure the degree of dissimilarity between movements performed in ‘real’ and ‘virtual’ scenarios. For this purpose, the 501 darts game and a video darts game played on a console were used. The participants’ arm throwing movements were video recorded and digitized. The matrices of *x* and *y* coordinates of the movements of the wrist, elbow, and shoulder in both performance scenarios were subjected to the Procrustes method. The wrist displays the most extreme dissimilarity values (higher than elbow and shoulder). Results also revealed smaller dissimilarity values for movements performed under the same conditions (e.g., real–real) and larger dissimilarity values between movements performed in different scenarios.

## Introduction

Nowadays, the use of simulators involving human movement to improve performance and learning is fairly common, with applications from motorsports to golf, from aircraft training pilots to motor rehabilitation. Furthermore, the technological improvement of recreational devices, such as game consoles, provides an easy contact with ‘virtual’ environments where participants need to move to play. The preciseness of movements required to succeed when performing game consoles tasks are closing the gap between movements performed in ‘virtual’ environments and movements performed in ‘real task’ scenarios. An effect of the subjectively closeness of human movement performance in these two scenarios is that simulators as game consoles or other virtual reality (VR) devices are being used to improve performance in different fields, such as sports or motor rehabilitation. However, the literature is still relatively reduced regarding methods that provide a quantitative analysis which measures the gap between a movement performed in a ‘virtual’ scenario and the same movement performed in a ‘real task’ scenario. Therefore, the aim of this article is to present a quantitative tool to measure the degree of dissimilarity between movements presented in different scenarios and to use it in a throwing darts task performed in a ‘real’ and a ‘virtual’ scenarios.

### Real and Virtual Task Scenarios

“The purpose of simulation is to reproduce some of the characteristics of a system or situation (e.g., sensory stimulation and constraints on behavior), without reproducing others (e.g., the expense and/or danger of operating the actual system)” (Stoffregen et al., 2003, pp. 114).

Every *virtual scenario*, from the most technological developed simulator as those used in the training of aircraft pilots to game consoles, aims to replicate sensory information and motor actions from ‘real’ scenarios. However, sensorial information duplication or movement repetition are impossible to achieve (Bernstein, 1967; Stoffregen et al., 2003). Visual and acoustic information that sustains subjects’ behavior is situation specific; in addition, subjects’ motion reciprocally influences the information that surrounds them (Gibson, 1979). The way that light reflects in surfaces and objects and the acoustic that is created within a performance context makes the information within that context unique; moreover, subjects’ motion has a direct influence on how the visual and acoustic information is perceived. This reciprocal influence between information and motion sustains that subjects’ behavior is situation specific. It was this hypothesis of *specificity* that led to behavioral differences between ‘real’ and ‘virtual’ performance scenarios (Gibson, 1979; Stoffregen et al., 2003).

Furthermore, inertial forces produced in simulated scenarios are different especially for motion-based simulators (e.g., game consoles as Xbox Kinect; flight simulators; F1 simulators). Within these ‘virtual’ scenarios, the inertial information has a lower magnitude, probably in a different direction and less duration when compared with ‘real’ scenarios, with behavioral consequences in the subjects’ movements (Riccio, 1995). This means that due to different loads of visual, acoustic, haptic, and vestibular information, subjects’ behavior when performing a motor task in a simulator is different when performing the same task in the system which intends to simulate.

Finally, differences in the sensory information between both scenarios (i.e., real and virtual) sustained on the hypothesis of specificity has consequences on subjects’ behavior. From the perspective of movement performance dissimilarity, these consequences still require further research. These behavioral differences expressed in participants’ movements dissimilarity can be suggested to quantify movement representativeness between both scenarios.

### Representative Design

The representative design, a concept introduced by Brunswik (1956), refers to a set of experimental task constraints so that they correspond to (or represent) the behavioral context to which the results of a research are intended to be generalized (Araujo et al., 2007; Pinder et al., 2011; Barris et al., 2013). This concept was adapted to performance and learning environments aiming to analyze how task constraints on practice settings are a faithful representation of competitive and/or performance contexts. Thus, the notion of “*representative learning design* refers to ensuring that the task constraints employed in training environments where learning may occur (e.g., during practice) are representative of those encountered by athletes in competitive performance context” (Barris et al., 2013, pp. 2).

Researchers’ main concern has been on how to measure the *degree of representativeness* between two (or even more) distinct scenarios as flight simulators and to fly a plane or team sports training exercises and to match sub-phases. More specifically, how to associate behavior in a practice setting with the performance (or competitive) setting which it is intended to generalize? This association has been performed through the *action fidelity* concept which aims to analyze the similarity of behavioral responses in different contexts (e.g., practice vs. competition; simulators vs. ‘real’) (Stoffregen et al., 2003; Araujo et al., 2007; Dicks et al., 2008; Pinder et al., 2011).

A previous study on diving from springboards meant to measure the dissimilarity (i.e., action fidelity) between diving in dry-land training environment and diving in aquatic competitive performance context (Barris et al., 2013). For that purpose, the authors searched for kinematics differences due to changes on interlimb coordination patterns and for differences in performance indicators as step lengths and jump heights on the springboard. The action fidelity was measured with plot diagrams of joint kinematics (e.g., angle-angle) of the same key events performed in dry-land and aquatic environments. Diagram shapes of joint kinematics were considered similar (i.e., topological equivalent) if one could be ‘stretched’ to form the other. The authors hypothesized that differences in topological patterns of movements performed in dry-land and in aquatic environments correspond to differences in coordination patterns when performing diving tasks under different constraints (Chow et al., 2008; Barris et al., 2013). Based on a qualitative analysis, data revealed topological similarities for all participants in the coordination patterns for both conditions (i.e., dry-land vs. aquatic) (Barris et al., 2013). The main achievement from the Barris and colleagues study was the development of a method based on joint kinematics and performance indicators to measure action fidelity. However, a quantitative analysis to measure similarities between two times series was still missing.

In sum, comparisons between outputs of ‘real’ and ‘virtual’ task scenarios have mainly been performed from a qualitative point of view. Here, we suggest that a powerful mathematical method of shape analysis, namely the Procrustes Analysis, can be used with data from ‘real’ and ‘virtual’ task scenarios to provide a quantitative approach for representativeness evaluation based on the dissimilarity of movement trajectories. It should be emphasized that the Procrustes Analysis method already exists, but in the current study it is -used in a new realm - the realm of movement analysis. In this exploratory study, we seek to quantify the movement representativeness in terms of dissimilarity between ‘real’ and ‘virtual’ motor performance scenarios.

### VR Environments and Motor Rehabilitation

In the literature, it has been suggested that motor skills can be learned through VR devices and one particular area of interest with application of VR systems is motor rehabilitation (Burke et al., 2009; Saposnik et al., 2010; Arya et al., 2011). This is a new and useful technology that allows users to interact with a computer generated scenario, therefore, a form of non-immersive VR. Virtual interfaces as game consoles allow users to move about and interact with virtual objects or virtual subjects in ways that are potentially more engaging than methods afforded by the traditional desktop environment. In particular, these technologies enable the observation of interactive avatar movements captured on screen and combine resources that induce adaptive changes and thus improve the performance of a motor skill (Burke et al., 2009; Saposnik et al., 2010). Moreover, VR training is advantageous because of the ability to simulate real-world environments that are too dangerous or expensive to replicate in the real world (e.g., training medical doctors to perform surgical procedures). The VR devices allow for increased volume and intensity of practice, while providing augmented three-dimensional and direct sensorial (visual, sensory, and auditory) feedback, with positive consequences in the learning of motor tasks (e.g., with patients involved in motor rehabilitation programs) (Holden and Dyar, 2002; Jang et al., 2005).

Previous research using game consoles, such as the Nintendo^®^Wii and Microsoft Xbox Kinect and other VR devices with computer games, in the motor rehabilitation of patients with Parkinson disease (Pompeu et al., 2014) and stroke patients (Jang et al., 2005; You et al., 2005; Pompeu et al., 2014), revealed functional motor gains. Concerning the experimental design, previous research with the use of VR devices in motor rehabilitation demonstrated that 60–90 min of VR intervention, three sessions/week, during 4–10 weeks is effective in obtaining measurable motor recovery in stroke patients (Holden and Dyar, 2002; Broeren et al., 2004). We hypothesize that this wide range of 4–10 weeks to obtain functional gains in motor recovering can be related to the representativeness of the VR devices used. However, how to measure the representativeness of an action performed in a VR device is still a gap in the literature.

Pointing to increase the man-machine interactive behavior, Microsoft developed the Kinect device to be used with the Xbox 360 game console, which is a device that captures the motion of the participant full body being unnecessary to use a device manually controlled. The Kinect device uses an infrared depth-sensing camera system to track the participant full body movements, and an embedded software recreates an avatar of the participant within the screen, so that all the participant movements are replicated by the avatar with an imperceptible delay (Lange et al., 2011).

But, as previously stated, representativeness is dependent upon the level of ‘realism’ created by the ‘virtual’ environment and can be constrained by the quality of the visual, auditory, and tactile feedback and length of exposure (Waller et al., 1998). From this perspective, the concept of representative design has recently been advocated to ensure that learning tasks are representative of the performance environment and may provide some insights of relevance for the association between practice and performance contexts that should be analyzed by considering the similarity of the participants’ actions. Motor learning is grounded on the interaction between the intrinsic dynamics of a participant and the demands of task and environmental constraints. We assume that task and environmental constraints in VR devices are different from those found in the context that they try to simulate, which means that an action performed in ‘virtual’ environments is in some degree different from the ‘same’ action performed in ‘real’ context. The main issue is on how to quantify this difference in motor actions. To quantify these differences will help to set criteria to guide the use of VR games as a therapeutic tool.

Therefore, the main objective of this study was to test the relevance of the Procrustes method to measure movement representativeness between ‘real’ and ‘virtual’ scenarios. Moreover, this is an exploratory study that intends to quantify the movement dissimilarities while performing a motor skill in a ‘real’ and in a ‘virtual’ environment.

## Materials and Methods

### Tools and Devices

For data collection, each participant was asked to play the darts game called 501 and to play a video game. For the 501 darts game, a set of three darts and a target was used. For the video game, a game console *Xbox 360 (Microsoft^®^*) was used with the *Kinect* device which has an RGB camera plus an infrared lighting. The selected game was the *Kinect Sports Darts*. An avatar was customized according to the participants’ characteristics of gender and physical appearance. These devices were connected to a 26-inch LCD TV and the *Kinect* was placed below the TV screen. Prior to data collecting, the procedures of both games were briefly explained. The target and the TV screen were placed at 1.20 m height from the floor and the participants stood at 2 m distance from both these equipment.

Each participant movements were captured using a single video camera (Casio Exilim 200ZR) which recorded all the trials at 30 Hz. The plane of motion was perpendicular (i.e., close to 90°) to the camera placement. For image treatment Kinovea 8.15 software was used which allows plotting the bi-dimensional coordinates (*x*, *y*) of the three points under analysis: (i) one located on the wrist; (ii) one located on the elbow; and (iii) one located on the shoulder of the participants.

### The Task

The task was performed in two different scenarios: (i) a ‘real’ environment where the participants were asked to perform a motor task with the dominant arm; the task required grasping a dart, pointing to the target, and then throwing the dart; the participants stood in front of the target 2 m away; (ii) a ‘virtual’ environment where the participants were instructed to play a video game which required performing a motor task with the dominant arm; the game was user friendly and the motor task of playing the game only required pointing to the target and corresponded to a movement similar to throwing the darts; the participants remained standing in front of the *Kinect* sensor at least 2 m away from the TV screen. For both scenarios, the goal was to reset the score of 501 points. For that purpose, the participants had to hit the target and the corresponding score was continuously decreasing the initial score of 501 points.

### Participants

The study included eight participants, healthy males (mean age = 23; *SD* = 8), without experience in video games. All the participants were voluntary and signed an informed consent form. The Ethics Committee of the Faculdade de Motricidade Humana, Universidade de Lisboa approved the study that was conducted according to the principles expressed in the Declaration of Helsinki. Throughout the text, the results from participant S0 will be used to exemplify the outputs of the Procrustes method and to highlight the pertinence of this technique to quantify movement dissimilarity in a specific motor task repeatedly performed in a ‘real’ and a ‘virtual’ scenarios. The results from all the participants will then be presented in terms of mean values.

### Experimental Design

Each participant performed alternatively four sets of three trials in the ‘real’ environment of throwing darts at a target and four sets of three trials in the ‘virtual’ videogame of Sports Darts played in the *Microsoft Xbox Kinect* device.

### The Procrustes Analysis

Procrustes Analysis is a mathematical method of comparing two shapes X and Y (matrices X and Y) based on determining a linear transformation (scaling, rotation, reflection, and translation) of the points in shape Y (matrix Y) to best match them to the points in shape X (matrix X). More precisely, for the comparison of two shape matrices X and Y with size nxp, where *n* is the number of points and p is the number of measurements per point, this method computes a transformed shape matrix *Z* with size nxp given by *Z* = *b Y R* + *C*, where *b* is a scaling factor that shrinks (*b* < 1) or stretches (*b* > 1) the shape, *R* is an orthogonal rotation and reflection matrix with size pxp, and *C* is a translation matrix with size nxp. In the transformation equation, the elements *b*, *R*, and *C* are selected to minimize the distance between the target shape matrix X and the transformed shape matrix *Z* measured by the sum of squared deviations. The dissimilarity measure between the two shapes is the minimized value of the sum of squared deviations standardized by the sum of squared elements of the mean centered target shape. The value of this measure lies between 0 and 1, with a value near 0 representing strong shape similarity and a value near 1 representing strong shape dissimilarity (Dryden and Mardia, 1998; Gower and Dijskterhuis, 2004). This value may also be presented in the form of a percentage, i.e., 0% for strong similarity and 100% for strong dissimilarity.

Procrustes Analysis may be used in numerous kinds of data to identify the disparity of specific features. In biological data, for instance, it can be used to quantify the variation of morphological elements or genetic components (Wang et al., 2010; Domjanic et al., 2013). In this study, this mathematical methodology was undertaken to truthfully quantify the difference of motor performances between ‘real’ and ‘virtual’ environments for the task of throwing darts. To overcome the problem of different lengths (number of distance points) of some pairs of real-virtual data sets, a linear interpolation method was used to match the smallest size to the largest one, by filling the largest empty spaces. This interpolation process was selected because it seemed not to modify the shape of the original data set.

### Simulation Study

The following section presents a simulation study to evaluate the properties of the Procrustes method and the reliability of its results. The selected matrix size was nxp = 30 × 2, where *n* = 30 resembles the mean length of the experimental series and *p* = 2 represents *x* and *y* coordinates. First, the target shape matrix X was simulated using a 4th order polynomial plus a Gaussian white noise (to be similar to some experimental series, e.g., of the shoulder). Next, the second shape matrix Y was taken as being equal to: (i) matrix X (*Y* = *X*); (ii) a scaled version of X (*Y* = *b*_0_ *X*, where *b*_0_ was 0.5); (iii) a rotated version of X (*Y* = *X R*_0_, where *R*_0_ was a rotation matrix with angle 10°); and (iv) a translated version of X [*Y* = *X* + *C*_0_, where *C*_0_ was a translation vector with direction (0.1, 1.0)]. Then, the Procrustes method was undertaken to best match each shape Y to the shape X and to obtain the corresponding values of the dissimilarity measure, as well as the scaling, rotations, and translations parameters.

**FIGURE 1:**
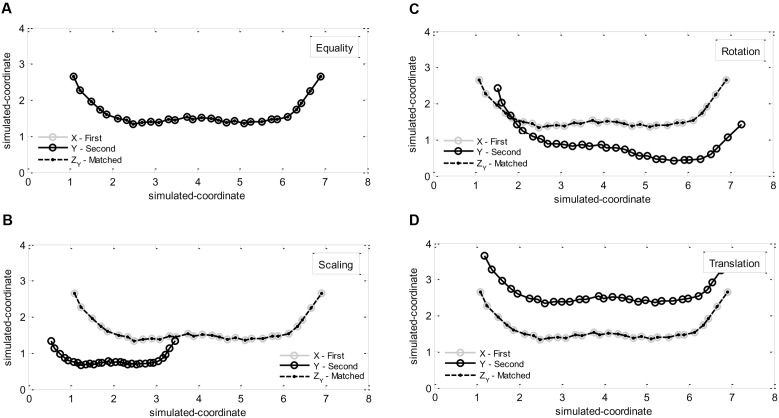
**Procrustes method for X and Y simulated shapes when shape Y is equal to:** **(A)** shape *X*, **(B)** a scaled version of X (by 0.5), **(C)** a rotated version of X (by 10°), **(D)** a translated version of X [by (0.1, 1.0)].

**Figure 1** exhibits the geometrical representations of the shapes X and Y and the Procrustes results for the four situations. In the first simulation (*Y* = *X*), as expected, the dissimilarity value was *d* = 0.0 (0.0%), the scaling factor was *b* = 1.0, the rotation matrix was *R* = identity matrix, and the translation vector was *C* = null vector. This output establishes a baseline, i.e., point of reference for further comparisons between two shapes. In the second simulation (*Y* = *b*_0_ *X*), the dissimilarity value was *d* = 0.0 (0.0%), the scaling factor was *b* = *b*_0_^-1^ = 2.0, the rotation matrix was *R* = identity matrix, and the translation vector was *C* = null vector. Moreover, in the third simulation (*Y* = *X R*_0_), the dissimilarity value was *d* = 0.0 (0.0%), the scaling factor was *b* = 1.0, the rotation matrix was *R* = *R*_0_^-1^ = rotation matrix with angle -10°, and the translation vector was *C* = null vector. Finally, in the fourth simulation (*Y* = *X* + *C*_0_), the dissimilarity value was *d* = 0.0 (0.0%), the scaling factor was *b* = 1.0, the rotation matrix was *R* = identity matrix, and the translation vector was *C* = -*C*_0_ = translation vector with direction (-0.1, -1.0). These results are reassuring for the possibility of using the Procrustes method to reliably quantify dissimilarity between two shapes obtained in different scenarios (e.g., movements performed in ‘real’ and ‘virtual’ task scenarios).

## Results

**FIGURE 2:**
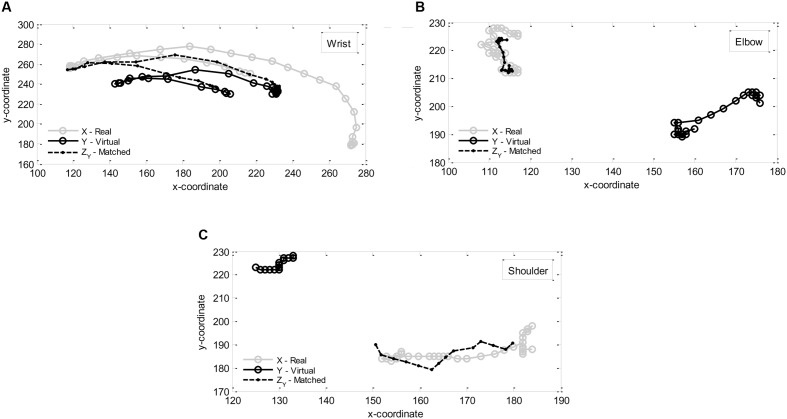
**Procrustes method for movement trajectories of participant S0 in real and virtual scenarios regarding:** **(A)** the wrist, **(B)** the elbow, **(C)** the shoulder in trial 3.

**Figure 2** illustrates, for participant S0, the Procrustes method applied to the pairs of shapes regarding the movements in the ‘real’ and ‘virtual’ scenarios of the wrist, the elbow, and the shoulder in trial 3. In this particular situation, scaling, rotations, and translations were performed in order to best match each ‘virtual’ shape to the related ‘real’ shape. The obtained dissimilarity value *d* (in percentage) between the movements performed in the ‘real’ and ‘virtual’ scenarios of the wrist was 57.7 [the scaling factor *b* was 1.3, the rotation matrix *R* had angle 3.5°, and the translation vector *C* had direction (-89.8, -48.2)]. The dissimilarity value *d* between both movements of the elbow was 26.0 [the scaling factor *b* was 0.5, the rotation matrix *R* had angle -63.9°, and the translation vector *C* had direction (165.8, 100.8)]. The dissimilarity value *d* between both movements of the shoulder was 18.8 [the scaling factor *b* was 3.1, the rotation matrix *R* had angle 30.3°, and the translation vector *C* had direction (-531.7, -211.4)]. These results show that the dissimilarity between the movement trajectories in the ‘real’ and ‘virtual’ scenarios of the wrist was larger than the one of the elbow, which in turn was larger than the one of the shoulder in this trial.

**FIGURE 3:**
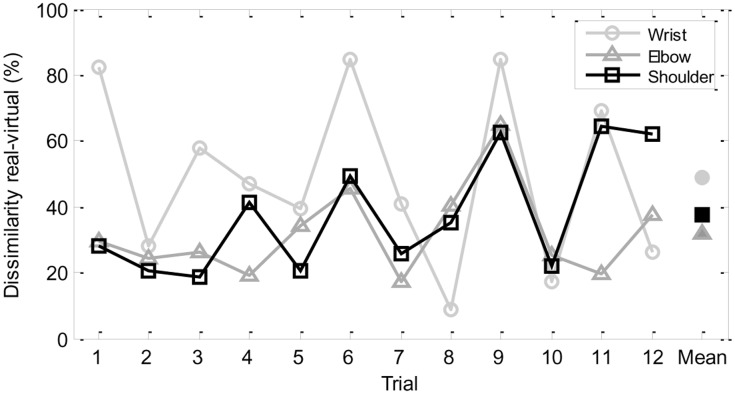
**Dissimilarity measure (percentage) between movement trajectories of participant S0 in real and virtual scenarios for the wrist, the elbow, and the shoulder across trials**.

**Figure 3** displays, for participant S0, the values of the dissimilarity measure (in percentage) between the movements in the ‘real’ and ‘virtual’ scenarios of the wrist, the elbow, and the shoulder in the successive trials performed, as well as the corresponding means over all trials. It can be seen that the highest peaks of dissimilarity between ‘real’ and ‘virtual’ scenarios (in trials 1, 6, 9, and 11), but also the lowest value of dissimilarity (in trial 8), correspond to wrist trajectories. Furthermore, the wrist trajectories attained the highest dissimilarity values when compared with the elbow and the shoulder trajectories in each trial, with the exception of trials 8, 10, and 12; on the other hand, the elbow movements achieved the lowest values of dissimilarity in trials 4, 6, 7, and 11, whereas the shoulder movements attained the lowest values of dissimilarity in trials 1, 2, 3, 5, and 9.

Next, for each participant, descriptive statistics of central tendency and dispersion were obtained for the dissimilarity values of the wrist, the elbow, and the shoulder movements over the trials. Then, taking into consideration the values of these statistics for all the participants, mean values were reported. The results show that the wrist is the element with the most extreme values, i.e., the largest mean, standard deviation, minimum, maximum, and range, together with the smallest coefficient of variation; on the other hand, the elbow and the shoulder present less extreme and closest values (**Table 1**). This reflects the occurrence of a higher dissimilarity between ‘real’ and ‘virtual’ scenarios for the wrist movements then for the elbow and the shoulder movements (see mean, minimum, and maximum values in **Table 1**). Simultaneously, there is a higher variability on the wrist movements over the trials when compared to the elbow and the shoulder movements (see standard deviation, and range values in **Table 1**).

**Table 1:** Mean descriptive statistics of the dissimilarity measure between movement trajectories in real and virtual scenarios over the trials.

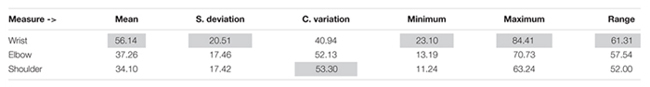

*Gray cells indicate the largest value in each column.*

In order to quantify the ‘internal similarity’ of the movements performed in ‘real’ scenarios, as well as the movements performed in ‘virtual’ scenarios, across trials, the Procrustes method was also applied to each pair of consecutive movement trajectories in ‘real’ scenarios and each pair of consecutive movement trajectories in ‘virtual’ scenarios. Then, descriptive statistics were obtained for the dissimilarity values of the real–real movement scenarios and the virtual–virtual movements scenarios. The outputs reveal once again that the wrist is the element with the most extreme statistical values, while the elbow and the shoulder present smaller values (with a few exceptions), in both conditions: movement trajectories in real–real scenarios (**Table 2**) and movement trajectories in virtual–virtual scenarios (**Table 3**). Moreover, the mean dissimilarity values of the wrist, the elbow, and the shoulder in the real–real movement scenarios are smaller than the ones in the virtual–virtual movement scenarios, which in turn are smaller than the ones in the real-virtual movement scenarios (**Table 1**); in parallel, the minimum and maximum of the dissimilarity values attain the smallest observations in the real–real movement scenarios and the virtual–virtual movement scenarios.

**Table 2:** Mean descriptive statistics of the dissimilarity measure between successive movement trajectories in real scenarios over the trials.

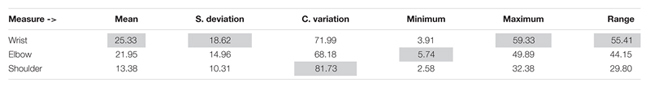

*Gray cells indicate the largest value in each column.*

**Table 3:** Mean descriptive statistics of the dissimilarity measure between successive movement trajectories in virtual scenarios over the trials.

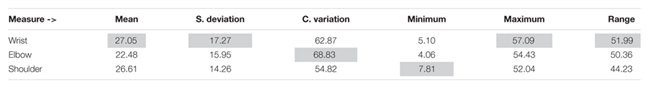

*Gray cells indicate the largest value in each column.*

## Discussion and Conclusion

This exploratory study suggests that the Procrustes analysis, used in the realm of movement analysis, is a suitable tool to reliably quantify dissimilarity and thus representativeness between movement trajectories in two distinct scenarios, as ‘real’ environments and ‘virtual’ environments. In fact, the simulation results highlight the capacity of the method to detect scaling, rotations, and/or translations to best match one shape to the other and then to obtain the dissimilarity value. This may be very useful for the precise evaluation of movement performance dissimilarity, particularly in sports performance, motor rehabilitation, medical surgical contexts, and other fields related to motion evaluation. One of the most important features of the utilization of this method in the realm of movement analysis is that it can be used to quantify dissimilarity in situations in which there are movement trajectories performed in different scenarios or performed by different subjects, for instance the various trajectories of a bike rider on a track, the several movement paths of a rehabilitation patient when lifting an object, or even the flight paths of two divers.

The present results revealed that distinct components of the same movement can have different values of dissimilarity. One example concerns the different (usually larger) dissimilarity values of the wrist trajectories regarding the elbow and the shoulder trajectories in all trials. A possible explanation is the existence of more degrees of freedom for the wrist movements than for the elbow and the shoulder movements, which affords the wrist the ability to produce movement readjustments to achieve the task goal. Furthermore, the same component (i.e., wrist; elbow; shoulder) can have different values of dissimilarity in different trials. This result may be due to the relatively small number of trials performed, which seems to imply that these trials were not enough to make the movement pattern reach stabilization. We hypothesize that increasing the number of trials may lead to learning effects and thus to a decrease in the variability of the dissimilarity values.

Finally, the smaller dissimilarity values for the movements performed in the real–real scenarios reinforce the trustworthiness of the Procrustes method when used in the realm of movement analysis. In reality, this output is in line with the specificity hypothesis (Gibson, 1979), which states that sensory information that surrounds the subject is situation specific. In the virtual scenario, the ‘throwing’ movement was performed without throwing an object and thus in the absence of relevant information (such as the weight and the thickness of the dart). As a consequence, the performer needed to adapt the ‘throwing’ movement to the lack of this relevant information. Therefore, due to the difference between the information surrounding the performer in the two scenarios (real; virtual), it was expected that the dissimilarity values obtained in the real–real scenarios were smaller than the ones of the real-virtual scenarios.

In conclusion, an interesting issue for future research with the Procrustes analysis using videogames or other VR devices is to relate the performer learning effects with the movement dissimilarity values. When the motor task performance tends to stabilize due to learning effects, the dissimilarity values between ‘real’ movement scenarios and ‘virtual’ movement scenarios, as well as their variability, will tend to decrease or to increase?

## Author Contributions

We certify that all authors have read the manuscript, the paper has not been previously published, and it is not under simultaneous consideration by another journal; also there is no ghost writing by anyone not named on the author list. Regarding the authorship all the authors provided substantial contributions to the conception, design, acquisition, and analysis as well as interpretation of data for the work. All authors drafted the work or revising it critically for important intellectual content and provided a Final approval of the version to be published. Finally all gave the agreement to be accountable for all aspects of the work in ensuring that questions related to the accuracy or integrity of any part of the work are appropriately investigated and resolved.

## Conflict of Interest Statement

The authors declare that the research was conducted in the absence of any commercial or financial relationships that could be construed as a potential conflict of interest.
